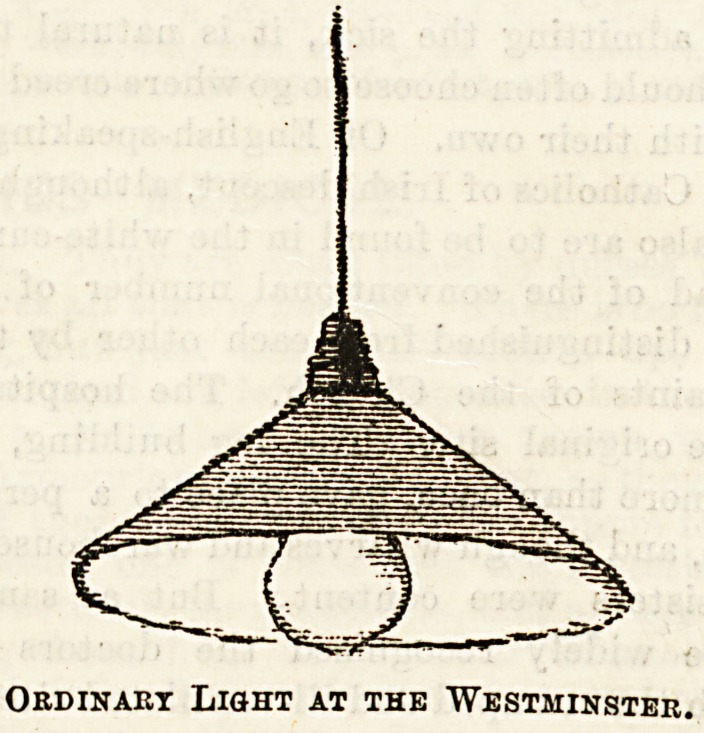# Electric Lighting in Hospitals

**Published:** 1894-10-27

**Authors:** 


### PRACTICAL DEPARTMENTS.

### ELECTRIC LIGHTING FOR HOSPITALS.

The use of the electric light in public buildings and institutions gains ground steadily, if slowly. In all new buildings of any size and importance it is becoming a matter of course that the system shall be installed from the beginning; but when it is a question of adaptation to existing institutions the balancing of expense against convenience gives matter for much deliberation, and too often hospital authorities are frightened to embark upon improvements, however important, when a very large initial expenditure is entailed. With the money difficulty once overcome, there cannot be two opinions as to the enormous advantage to be gained by its adoption.

The larger London hospitals are introducing the system of electric lighting slowly, as funds—and their respective Boards—will permit. Middlesex Hospital is, so far as we are aware, the only one among them to have put in the light all over the building, from basement to roof. This being so our readers may perhaps find some interest in a few particulars as to the comparative cost and the general success of the enterprise, which, by the courtesy of Mr. Melhardo, the secretary-superintendent, we are enabled to give. We select Middlesex Hospital for special comment, because the installation has there been so completely carried through. Of other general hospitals, where the lighting has been partially carried out we shall speak later.

Ordinary Light at the Westminster.

The work at the Middlesex Hospital was entrusted to Mr. T. H. White, of 9 and 11, Mill Street, Regent Street, and the current is obtained from the Metropolitan Electric Light Company at a cost of 7d., per Board of Trade unit, which is reduced to 5d. on all consumption over 2,000 units. The original cost of the installation throughout every part of the hospital proper, the Nurses' Home, Medical School, Trained Nurses' Institute, &c. (1,147 lights in all) was £2,863 11s. 2d. The cost of wiring, and providing switches and terminals came to 30s. per light, but many of the fittings, such as those in the out-patient department, in the ophthalmic and aural rooms, were of a more expensive kind, and were charged in addition. Unfortunately the cost of installation is not the only expense to be borne in mind when contemplating the adoption of the electric light. It is also more expensive to maintain, though the difference between the cost of consumption of gas and electric light is more apparent than real, and by no means so great as a superficial examination of the figures would seem to indicate. Taking the years 1889 and 1893 for comparison (these years are selected because, though the hospital was not entirely illuminated with electric light till 1893, the installation was begun in 1890), Mr. Melhardo estimates the actual increase on expenditure in lighting for nine months in each year at £696 18s. 5d., a sum which certainly at first seems somewhat heavy. But against this many counterbalancing advantages have to be placed, not the least among these being a considerable economy in cleaning, whitewashing, &c. Mr. Whitely has, we believe stated that he estimates the saving effected in general cleaning on his premises since the electric light has been in use there at £500 a-year, and though in hospitals, for sanitary reasons, the saving on this head cannot be quite the same as in private establishments, it is undoubtedly a point for consideration. It has also to be remembered that the number of lights is far more numerous than in the days of gas, quite "tenfold that of the former number of gas burners."

The wards at the Middlesex Hospital are well supplied with lights. Of these Miss Thorold, the Matron, has very kindly allowed a few sketches to be taken, which we shall give by way of illustration. We have, however, only space this week, for the representation of one of the ordinary lights, with opaque shades, which are suspended down the centre of the wards.

(*To be continued.*)

## Figures and Tables

**Figure f1:**